# Edoxaban versus enoxaparin for the prevention of venous thromboembolism after total knee or hip arthroplasty: pooled analysis of coagulation biomarkers and primary efficacy and safety endpoints from two phase 3 trials

**DOI:** 10.1186/s12959-016-0121-1

**Published:** 2016-12-01

**Authors:** Yohko Kawai, Takeshi Fuji, Satoru Fujita, Tetsuya Kimura, Kei Ibusuki, Kenji Abe, Shintaro Tachibana

**Affiliations:** 1 International University of Health and Welfare, 8-10-16 Akasaka, Minato-ku, Tokyo 107-0052 Japan; 2 Department of Orthopaedic Surgery, Japan Community Healthcare Organization Osaka Hospital, 4-2-78, Fukushima, Fukushima-ku, Osaka 553-0003 Japan; 3 Department of Orthopaedic Surgery, Takarazuka Daiichi Hospital, 19-5 Kogetsu-cho, Takarazuka, 665-0832 Japan; 4 Daiichi Sankyo Co., Ltd, 3-5-1, Nihonbashi Honcho, Chuo-ku, Tokyo 103-8426 Japan; 5 Clinical Data & Biostatistics Department, Daiichi Sankyo Co. Ltd, 1-2-58, Hiromachi, Shinagawa-ku, Tokyo 140-8710 Japan; 6 Department of Orthopaedic Surgery, Mishuku Hospital, 5-33-12 Shimomeguro, Meguro-ku, Tokyo 153-0051 Japan

**Keywords:** DOAC, Total knee arthroplasty, Total hip arthroplasty, Biomarker, VTE prophylaxis

## Abstract

**Background:**

The objective of this analysis was to assess the effects of edoxaban compared with enoxaparin on key coagulation biomarkers and present pooled primary efficacy and safety results from phase 3 STARS E-3 and STARS J-V trials for prevention of venous thromboembolism (VTE) after total knee arthroplasty (TKA) or total hip arthroplasty (THA).

**Methods:**

In the randomized, double-blind, double-dummy, multicenter, STARS E-3 and STARS J-V trials, patients received edoxaban 30 mg or enoxaparin 2000 IU (20 mg) twice daily for 11 to 14 days. The studies were conducted in Japan and Taiwan; enoxaparin dosing was based on Japanese label recommendations. The primary efficacy endpoint was incidence of VTE; the safety endpoint was major or clinically relevant nonmajor (CRNM) bleeding. Blood samples were taken at presurgical evaluation, pretreatment (postsurgery), predose on day 7, predose on completion of treatment, and at a follow-up examination 25 to 35 days after the last dose of study drug for D-dimer, prothrombin fragment 1 + 2 (F_1+2_), and soluble fibrin monomer complex (SFMC) measurement.

**Results:**

A total of 716 patients enrolled in STARS E-3 and 610 patients enrolled in STARS J-V; 1326 patients overall. This analysis included 657 patients who received edoxaban 30 mg QD and 650 patients who received enoxaparin 20 mg BID. Incidence of VTE was 5.1 and 10.7% for edoxaban and enoxaparin, respectively (*P* <0.001). Incidence of combined major and CRNM bleeding was 4.6 and 3.7% for edoxaban and enoxaparin, respectively (*P* = 0.427). On day 7, mean D-dimer (4.4 vs 5.5 μg/mL), F_1+2_ (363 vs 463 pmol/L), and SFMC (5.7 vs 6.8 μg/mL) were lower in edoxaban-treated patients relative to enoxaparin-treated patients, respectively (*P* <0.0001 for all). At end of treatment, mean D-dimer (5.4 vs 6.2 μg/mL), F_1+2_ (292 vs 380 pmol/L), and SFMC (6.2 vs 7.2 μg/mL) were lower in edoxaban-treated patients relative to enoxaparin-treated patients (*P* <0.0001 for all).

**Conclusions:**

Edoxaban was superior to enoxaparin in prevention of VTE following TKA and THA, with comparable rates of bleeding events. Relative to enoxaparin, edoxaban significantly reduced D-dimer, F_1+2_, and SFMC.

**Trial registration:**

Clintrials.gov NCT01181102 and NCT01181167. Both registered 8/12/2010.

**Electronic supplementary material:**

The online version of this article (doi:10.1186/s12959-016-0121-1) contains supplementary material, which is available to authorized users.

## Background

Patients undergoing orthopedic surgery such as total knee arthroplasty (TKA) or total hip arthroplasty (THA) are at high risk for venous thromboembolism (VTE) [1, 2]. Anticoagulation therapy and/or mechanical prophylaxis, including compression stockings or intermittent pneumatic compression, are recommended for prevention of VTE after orthopedic surgery [1, 2]. In Japan, edoxaban [3], a direct oral anticoagulant (DOAC) selective inhibitor of activated factor Xa (FXa), and enoxaparin [4], an injectable low-molecular-weight heparin (LMWH), are both indicated for prophylaxis of deep vein thrombosis (DVT) following TKA, THA, or hip fracture surgery. The approval of edoxaban for the primary prevention of VTE after lower limb orthopedic surgery was based on evidence collected during three phase 3 studies evaluating the safety and efficacy of edoxaban compared with enoxaparin for prevention of VTE in Japanese or Taiwanese patients following TKA [5], THA [6], and hip fracture surgery [7]. In these studies, edoxaban demonstrated significantly reduced or comparable rates of VTE and similar rates of bleeding events relative to enoxaparin.

This report presents a post hoc pooled analysis of coagulation biomarkers in the TKA/THA studies as well as pooled results of the primary efficacy (VTE) and safety (bleeding events) endpoints. Coagulation biomarkers include D-dimer, prothrombin fragments 1 + 2 (F_1+2_), and soluble fibrin monomer complex (SFMC). D-dimer, which has a high negative predictive value for VTE, is formed upon cleavage of cross-linked fibrin polymers by plasmin [8–10]. F_1+2_ is a marker of thrombin generation and represents coagulation activity [11]. Fibrin monomers result from cleavage of fibrinogen by thrombin [8]. Soluble fibrin in plasma is also a marker of coagulation activity and is seen to increase rapidly during and after hip replacement surgery [12]. Assessment of coagulation biomarkers can provide information on the effect of anticoagulants in relation to dose and clinical response.

## Methods

Detailed descriptions of the methodology of these trials are available in the primary publications (STARS E-3 [5] and STARS J-V [6]). The trial designs for patients undergoing TKA (STARS E-3; NCT01181102) or THA (STARS J-V; NCT01181167) were similar. In the randomized, double-blind, double-dummy, multicenter trials, patients received oral edoxaban 30 mg or edoxaban placebo once daily within 6 to 24 h after surgery, and subcutaneous enoxaparin 2000 IU (equivalent to 20 mg) or enoxaparin placebo twice daily within 24 to 36 h after surgery, each for 11 to 14 days. Enoxaparin 20 mg is the usual recommended dose for adults in Japan due to the lower body weight of Japanese patients [13]; standard of care is administration of enoxaparin 24 to 36 h postsurgery.

Concomitant use of anticoagulants, antiplatelet agents, thrombolytic agents, or other agents that affect thrombus formation was not allowed from the day of surgery until 24 h after the final dose of study drug, unless treatment of deep vein thrombosis or pulmonary embolism (PE) was required. Mechanical prophylaxis (eg, elastic stockings or intermittent pneumatic compression therapy of the foot sole or lower leg and thigh) was permitted from the day of surgery to venography. Venography of the operated lower limb in the TKA trial STARS E-3 and of both lower limbs in the THA trial STARS J-V was performed within 24 h of the last dose of study drug or within 96 h in exceptional cases such as difficulty establishing an intravenous line.

The studies were performed in accordance with the provisions of the Declaration of Helsinki, Guidelines for Good Clinical Practice, and other related regulations. The protocols were approved by institutional review boards at each study center, and written informed consent was obtained from all patients prior to randomization.

### Patients

Men and women 20 to <85 years of age undergoing unilateral TKA or THA (both excluding revision arthroplasty) were included. Presurgical exclusion criteria included risk for bleeding, risk for thromboembolism, previous TKA, weight <40 kg, severe renal impairment (creatinine clearance <30 mL/min) [14], evidence of hepatic dysfunction (serum aspartate aminotransferase or serum alanine aminotransferase levels ≥2 times the upper limit of normal or total bilirubin ≥1.5 times the upper limit of normal), previous treatment with edoxaban, and current antithrombotic therapy for another complication. Postsurgical exclusion criteria included abnormal bleeding from the puncture site during spinal anesthesia, need for repeat surgery before the start of study treatment, abnormal or excessive bleeding experienced during surgery, and inability to take oral medication.

### Assessments

Thromboembolic events included asymptomatic or symptomatic DVT—confirmed by venography at the end of study treatment—and symptomatic and diagnosed PE. Additional imaging techniques used to confirm suspected DVT or PE included ultrasonography, computerized tomography scanning, pulmonary scintigraphy, or pulmonary arteriography.

Major bleeding was defined as fatal bleeding; clinically overt bleeding accompanied by a decrease in hemoglobin of >2 g/dL or requiring transfusion with >800 mL of blood; retroperitoneal, intracranial, intraocular, or intrathecal bleeding; or bleeding requiring repeat surgery. Clinically relevant nonmajor (CRNM) bleeding was defined as bleeding that did not meet the criteria for major bleeding, but was characterized by hematoma ≥5 cm in diameter, epistaxis or gingival bleeding in the absence of external factors lasting ≥5 min, gastrointestinal bleeding, gross hematuria persistent after 24 h of onset, or any other bleeding deemed clinically significant by the investigator. Minor bleeding was any bleeding event that was not considered a major or CRNM bleeding event. Thromboembolic events were assessed by the blinded Thromboembolic Event Assessment Committee and bleeding events by the Bleeding Event Assessment Committee.

Blood sampling was performed at presurgical evaluation, pretreatment (postsurgery), predose on day 7, predose on completion of treatment, and at a follow-up examination 25 to 35 days after the last dose of study drug. All biomarker assessments for D-dimer, F_1+2_, and SFMC were performed and measured at a central laboratory (SRL Inc., Tokyo, Japan). D-dimer was measured by a latex agglutination assay using the LATECLE D-dimer test kit (Kainos Laboratories, Inc., Tokyo, Japan; upper limit of detection, 1.0 μg/mL); data were expressed as D-dimer units. Assessment of F_1+2_ was performed via ELISA (Fibinostika, Organon Teknika BV, The Netherlands; normal detection range 69–229 pmol/L) [15] and assessment of SFMC was performed via a latex immunoturbidimetric assay (upper limit of detection, 6.1 μg/mL) [16].

Treatment compliance was assessed by clinical interview with patients and by remaining drugs collected.

### Statistical analysis

**Fig. 1:**
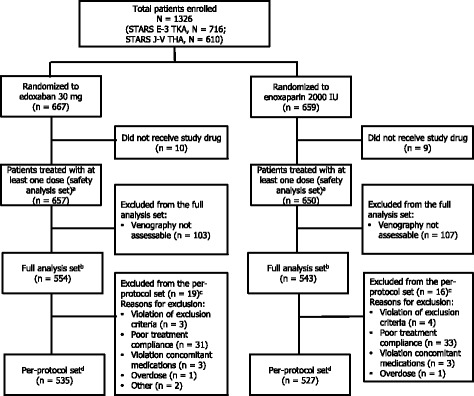
Distribution of patients in the pooled data analyses. ^*a*^The safety analysis set included all enrolled patients who received study drug, had posttreatment safety data, and did not have significant GCP violations. ^*b*^The full analysis set included all patients receiving ≥1 dose of study drug and excluded patients with significant GCP violations or with inadequate venography. ^*c*^Multiple answers were allowed; patients falling under multiple categories were counted once for each category. ^*d*^The per-protocol set included patients in the FAS and excluded patients with violations of inclusion or exclusion criteria, violation of rules for prohibited concomitant drugs/treatment, or <80% compliance with study drug. GCP = good clinical practice; FAS = full analysis set; THA = total hip arthroplasty; TKA = total knee arthroplasty

The primary efficacy endpoint—the proportion of patients who experienced at least 1 thromboembolic event from the start of treatment to venography—was assessed in the full analysis set of patients, those who received ≥1 dose of study drug and who underwent interpretable venography. Baseline data and safety results were analyzed in the safety set—patients who received ≥1 dose of study drug and had safety data collected after the start of treatment. Biomarker results were analyzed in the pharmacodynamic set—patients who received ≥1 dose of study drug, had no protocol violations, had compliance rates of ≥80%, and had ≥1 biomarker measurement (Fig. 1).

The number of VTE events and number of bleeding events across the 2 trials were added. The Farrington-Manning method [17] was used to derive the difference in VTE incidence. The SCORE method [18] was used to calculate 95% confidence intervals (CIs) for both VTE and bleeding events. For analysis of coagulation biomarkers, summary statistics were calculated by group and time.

Paired comparisons between groups were performed using chi squared or Wilcoxon rank sum testing with a significance level set to 5%. All statistical tests were conducted as 2-sided tests.

## Results

### Patients

There were no significant differences in baseline characteristics between the combined treatment groups from the 2 trials (Table 1). Overall, patients were predominantly women (83%) of a mean age of 68 years. The primary disease was most frequently osteoarthritis (88%). A total of 1326 patients were enrolled; this analysis included 657 patients who received edoxaban 30 mg once daily and 650 patients who received enoxaparin 20 mg twice daily. Patient disposition was similar between the 2 trials (Fig. 1).

**Table 1:** Patient demographics and baseline characteristics

Variable	Edoxaban 30 mg QD *N* = 657	Enoxaparin 20 mg BID *N* = 650	*P* value
Female, n (%)	552 (84.0)	527 (81.1)	0.161^a^
Age, years, mean (min–max)	68.3 (36–84)	68.1 (24–84)	0.760^b^
Body weight, kg, mean (min–max)	58.7 (40–124)	58.8 (40–98)	0.848^b^
Creatinine clearance, mL/min, mean (min–max)	82.1 (30.6–242.9)	81.7 (31.0–209.7)	0.804^b^
Primary disease, n (%)			
Osteoarthritis	582 (88.6)	563 (86.6)	0.270^c^
Rheumatoid arthritis	42 (6.4)	46 (7.1)	
Other	35 (5.0)	41 (6.3)	

*BID* twice daily, *QD* once daily

^a^Chi square test

^b^t test

^c^Wilcoxon test

### Primary efficacy endpoint

**Fig. 2:**
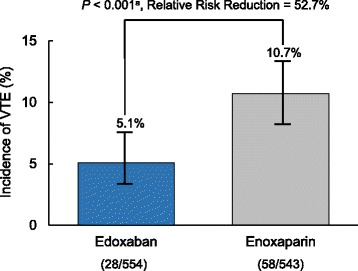
Primary efficacy endpoint – incidence of VTE. ^a^Chi square test. VTE = venous thromboembolism

The composite of asymptomatic DVT and symptomatic DVT or PE occurred in 28 of 554 patients who received edoxaban (5.1%) and 58 of 543 patients who received enoxaparin (10.7%), *P* <0.001 (Fig. 2). Thromboembolic events were primarily asymptomatic DVT.

### Biomarkers

**Fig. 3:**
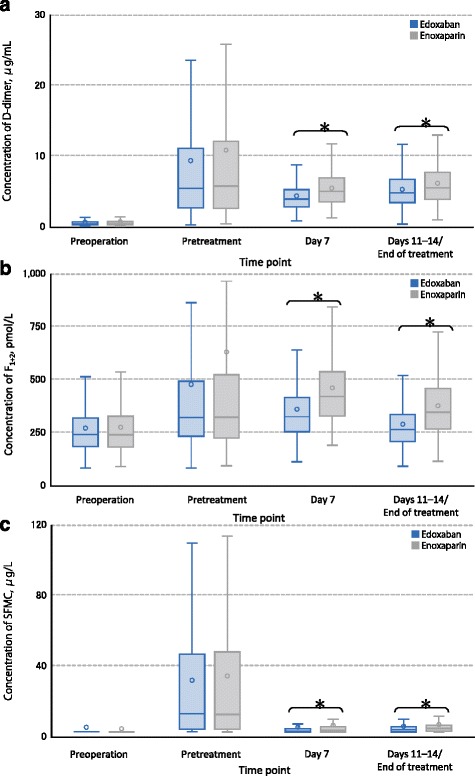
Levels of coagulation biomarkers. **a** D-dimer; **b** Prothrombin fragments 1 + 2 (F_1+2_); **c** Soluble fibrin monomer complex (SFMC). Open circles mark mean; horizontal lines indicate median; boxes represent 25–75%; capped lines represent 10 and 90%; ^*^ = *P* <0.001 (Wilcoxon test)

Plasma levels of the coagulation biomarker D-dimer are shown in Fig. 3a and Table 2. Mean D-dimer concentrations substantially increased after surgery but before treatment. After treatment, mean D-dimer levels (standard deviation [SD]) decreased significantly more in the edoxaban-treated than the enoxaparin-treated patients, respectively, both on day 7 (4.4 [2.1] vs 5.5 [2.6] μg/mL) and at the end of treatment (days 11–14) (5.4 [2.5] vs 6.2 [3.1] μg/mL), *P* <0.0001 for both. Median values and ranges are provided in Additional file 1: Table S1.

**Table 2:** Mean plasma concentrations of coagulation biomarkers at various time points after total knee or total hip arthroplasty

		Preoperation		Pretreatment		Day 7^a^		End of treatment (days 11–14)^a^	
		n	Mean (SD)	n	Mean (SD)	n	Mean (SD)	n	Mean (SD)
D-dimer (μg/mL)	Edoxaban	535	0.73 (0.82)	535	9.42 (12.56)	532	4.43^b^ (2.08)	528	5.37^b^ (2.52)
	Enoxaparin	527	0.78 (0.96)	527	10.92 (16.23)	480	5.53 (2.56)	472	6.23 (3.12)
F_1+2_ (pmol/L)	Edoxaban	535	273.9 (150.6)	535	479.7 (741.8)	532	362.8^b^ (164.2)	528	292.1^b^ (167.6)
	Enoxaparin	527	277.8 (160.9)	527	633.2 (3234.9)	480	463.3 (185.6)	472	379.6 (174.4)
SFMC (μg/mL)	Edoxaban	535	5.62 (17.86)	535	32.25 (40.47)	532	5.71^b^ (9.76)	528	6.15^b^ (10.72)
	Enoxaparin	527	4.81 (8.42)	527	34.72 (45.62)	480	6.82 (13.99)	472	7.23 (11.78)

*F*_*1+2*_ thrombin fragments 1 + 2, *SD* standard deviation, *SFMC* soluble fibrin monomer complex

^a^Predose

^b^ *P* vs enoxaparin <0.0001 (Wilcoxon test)

Mean F_1+2_ concentrations increased after surgery and decreased following treatment with edoxaban or enoxaparin. The observed decrease in F_1+2_ following edoxaban treatment was larger relative to the decrease observed with enoxaparin treatment (Fig. 3b and Table 2). The mean F_1+2_ concentrations (SD) in edoxaban-treated and enoxaparin-treated patients, respectively, on day 7 of treatment were 363 (164) vs 463 (186) pmol/L and at the end of treatment were 292 (168) vs 380 (174) pmol/L, *P* <0.0001 for both. Median values and ranges are provided in Additional file 1: Table S1.

Mean SFMC concentrations rose after surgery and showed a larger decrease following edoxaban treatment relative to enoxaparin treatment (Fig. 3c and Table 2). The mean SFMC concentrations (SD) in edoxaban and enoxaparin patients, respectively, on day 7 were 5.7 (9.8) vs 6.8 (14.0) μg/mL and at the end of treatment were 6.2 (10.7) vs 7.2 (11.8), *P* <0.0001 for both. Median values and ranges are provided in Additional file 1: Table S1.

Assessment of plasma concentrations of biomarkers was performed in patients stratified by the presence or absence of VTE and the presence or absence of major or CRNM bleeding. Values followed a similar trend for patients with and without VTE and for edoxaban and enoxaparin treatment for D-dimer and F_1+2_ (Table 3). Values for SFMC were similar between edoxaban and enoxaparin treatments and were numerically elevated for patients with VTE relative to those who did not have VTE. Values for D-dimer, F_1+2_, and SFMC followed a similar trend for patients with and without CRNM and for treatment with edoxaban and enoxaparin (Table 4).

**Table 3:** Mean plasma concentrations of coagulation biomarkers at various time points after total knee or total hip arthroplasty in patients with and without VTE

		Preoperation		Pretreatment		Day 7^a^		End of treatment (days 11–14)^a^	
		n	Mean (SD)	n	Mean (SD)	n	Mean (SD)	n	Mean (SD)
Patients without VTE									
D-dimer (μg/mL)	Edoxaban	526	0.73 (0.84)	526	9.33 (12.54)	521	4.40 (2.09)	511	5.35 (2.49)
	Enoxaparin	485	0.77 (0.94)	485	10.28 (14.82)	443	5.38 (2.32)	430	6.00 (2.96)
F_1+2_ (pmol/L)	Edoxaban	526	273.6 (150.3)	526	478.6 (748.6)	521	361.5 (164.6)	511	293.5 (169.3)
	Enoxaparin	485	273.9 (139.3)	485	614.6 (3357.3)	443	457.9 (183.8)	430	372.6 (166.6)
SFMC (μg/mL)	Edoxaban	526	5.38 (17.34)	526	31.21 (39.32)	521	5.55 (9.04)	511	6.31 (11.22)
	Enoxaparin	485	4.33 (6.04)	485	31.87 (43.53)	443	6.22 (11.68)	430	6.94 (10.80)
Patients with VTE									
D-dimer (μg/mL)	Edoxaban	28	0.75 (0.86)	28	8.40 (9.17)	24	4.56 (1.52)	23	5.46 (2.88)
	Enoxaparin	58	0.90 (0.97)	58	16.96 (24.17)	47	7.06 (3.86)	49	8.41 (3.78)
F_1+2_ (pmol/L)	Edoxaban	28	258.4 (117.4)	28	483.3 (220.5)	24	352.9 (128.3)	23	248.7 (86.31)
	Enoxaparin	58	309.8 (273.2)	58	824.8 (959.3)	47	531.2 (213.6)	49	444.5 (222.0)
SFMC (μg/mL)	Edoxaban	28	8.90 (21.23)	28	52.19 (48.89)	24	8.10 (18.70)	23	4.77 (2.38)
	Enoxaparin	58	8.36 (18.17)	58	63.67 (56.12)	47	12.31 (26.32)	49	9.85 (17.73)

*F*_*1+2*_ thrombin fragments 1 + 2, *SD* standard deviation, *SFMC* soluble fibrin monomer complex, *VTE* venous thromboembolism

^a^Predose

**Table 4:** Mean plasma concentrations of coagulation biomarkers at various time points after total knee or total hip arthroplasty in patients with and without major or clinically relevant nonmajor bleeding

		Preoperation		Pretreatment		Day 7^a^		End of treatment (days 11–14)^a^	
		n	Mean (SD)	n	Mean (SD)	n	Mean (SD)	n	Mean (SD)
Patients without major or CRNM bleeding									
D-dimer (μg/mL)	Edoxaban	627	0.75 (0.99)	627	9.75 (12.95)	597	4.43 (2.07)	578	5.38 (2.49)
	Enoxaparin	626	0.78 (0.92)	626	10.72 (15.39)	552	5.47 (2.53)	517	6.15 (3.00)
F_1+2_ (pmol/L)	Edoxaban	627	275.4 (148.0)	627	484.7 (736.0)	597	361.1 (162.2)	578	291.7 (163.2)
	Enoxaparin	626	276.4 (153.3)	626	617.3 (2975.3)	552	463.4 (192.4)	517	378.2 (171.4)
SFMC (μg/mL)	Edoxaban	627	5.72 (17.49)	627	32.69 (40.46)	597	5.66 (9.50)	578	6.30 (11.18)
	Enoxaparin	626	4.80 (8.14)	626	34.71 (45.40)	552	6.88 (13.49)	517	7.12 (11.43)
Patients with major or CRNM bleeding									
D-dimer (μg/mL)	Edoxaban	30	0.52 (0.27)	30	8.73 (11.84)	15	4.53 (1.70)	9	6.29 (3.52)
	Enoxaparin	24	0.89 (1.29)	24	8.95 (9.28)	14	5.24 (1.86)	10	8.40 (6.20)
F_1+2_ (pmol/L)	Edoxaban	30	264.5 (108.3)	30	440.3 (430.4)	15	371.5 (141.5)	9	325.0 (140.9)
	Enoxaparin	24	265.8 (113.4)	24	526.2 (714.4)	14	470.3 (143.3)	10	449.0 (120.6)
SFMC (μg/mL)	Edoxaban	30	3.41 (1.82)	30	30.28 (44.24)	15	4.03 (1.06)	9	6.42 (3.20)
	Enoxaparin	24	5.05 (4.38)	24	26.66 (32.86)	14	4.08 (1.49)	10	7.84 (4.31)

*CNRM* clinically relevant nonmajor, *F*_*1+2*_ thrombin fragments 1 + 2, *SD* standard deviation, *SFMC* soluble fibrin monomer complex

^a^Predose

### Safety

**Fig. 4:**
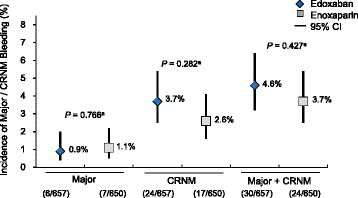
Incidence of major and CRNM bleeding events. ^*a*^Chi square test; CI = confidence interval; CRNM = clinically relevant nonmajor

There were no significant differences in the incidence of bleeding events during the trial between groups treated with edoxaban or enoxaparin (Fig. 4). Combined major and CRNM bleeding events occurred in 4.6% of edoxaban-treated and 3.7% of enoxaparin-treated patients (*P* = 0.427). The incidence of adverse events (AEs) was slightly lower in the edoxaban group (66%) than the enoxaparin group (75%). There were no differences in the frequency of serious AEs between the treatment groups [5, 6].

## Discussion

The risk of VTE increases after knee or hip arthroplasty [1, 2]. As shown in this pooled analysis of two phase 3 trials 11 to 14 days after surgery for TKA or THA, the incidence of VTE was significantly lower in patients administered once-daily oral edoxaban 30 mg (5.1%) than in those receiving twice-daily subcutaneous enoxaparin 20 mg (10.7%), *P* <0.001. Coagulation biomarkers D-dimer, F_1+2_, and SFMC each increased immediately after surgery. Over the course of 11 to 14 days, levels of the coagulation biomarkers were significantly lower after treatment with the DOAC edoxaban relative to the LMWH enoxaparin. In contrast, the frequency of bleeding events in the pooled results did not significantly differ.

Doses and timing used in this study are consistent with the Japanese standard of care for enoxaparin. Japanese patients typically have a lower body weight relative to their Western counterparts. Although the dose of enoxaparin used was low (2000 IU, twice daily), this is the recommended dose specific to Japan for prevention of VTE [4]. Prophylactic, subcutaneous enoxaparin doses of 40 mg once daily or 30 mg twice daily in males weighing >57 kg are associated with increased enoxaparin exposure and increased bleeding risk. Administration of LMWH 2 to 4 h postoperatively has been associated with higher rates of major bleeding relative to administration at 12 to 48 h postoperatively [19]. The Japanese standard of care calls for initiation of enoxaparin 24 to 36 h following surgery.

The results of STARS E-3 (TKA) [5] and STARS J-V (THA) [6] followed the same pattern as the pooled results reported here, with an incidence of VTE after surgery of 7.4 and 2.4% for edoxaban and 13.9 and 6.9% for enoxaparin in the 2 trials, respectively, and no significant differences in bleeding events. In a phase 2, dose-finding study in Japan, mean levels of D-dimer and F_1+2_ increased after TKA and remained above baseline for 11 to 14 days in placebo-treated patients, whereas treatment with edoxaban after surgery significantly reduced levels of the coagulation biomarkers in a dose-dependent manner [20]. In a retrospective study of patients undergoing TKA in Japan, patients treated with edoxaban 15 mg once daily showed significant reductions in D-dimer relative to enoxaparin 20 mg twice daily or fondaparinux 1.5 mg once daily over a 2-week period following surgery [21].

Edoxaban directly and selectively inhibits FXa, which is part of both the intrinsic and extrinsic coagulation pathways that lead to generation of thrombin and clot formation [22, 23]. One molecule of FXa can catalyze the formation of approximately 1000 thrombin molecules [23]. In contrast, LMWHs target FXa indirectly and affect multiple targets in the coagulation pathway [23]. The direct and selective targeting of FXa by edoxaban may account for the significantly greater reduction in coagulation biomarkers, which translates to reduced rates of VTE.

Limitations of this analysis include that it is post hoc and that it combines data from 2 different studies. However, the studies were very similar in anticoagulant treatment regimens and patient characteristics. In addition, for the coagulation biomarker results, pooling of results was required to obtain sufficient data to perform statistical comparisons between treatments. It also should be noted that edoxaban is approved only in Japan for VTE prophylaxis and is not approved for this indication in Europe or the United States.

## Conclusions

In conclusion, the biomarker results for the pooled analysis of the TKA and THA trials may suggest stronger anticoagulant activity with once-daily oral edoxaban 30 mg than twice-daily, subcutaneous enoxaparin 20 mg following lower limb orthopedic surgery, although the initial timing of edoxaban or enoxaparin administration differed. The 2 treatments were associated with similar rates of bleeding events.

## Additional file

Additional file 1: Table S1. Median and range of plasma concentrations of coagulation biomarkers at various time points after total knee or total hip arthroplasty. (DOCX 14 kb)

## Abbreviations

AE: Adverse event
CRNM: Clinically relevant nonmajor
DOAC: Direct oral anticoagulant
DVT: Deep vein thrombosis
F_1+2_: Prothrombin fragments 1 + 2
FXa: Factor Xa
LMWH: Low-molecular-weight heparin
PE: Pulmonary embolism
SD: Standard deviation
SFMC: Soluble fibrin monomer complex
THA: Total hip arthroplasty
TKA: Total knee arthroplasty
VTE: Venous thromboembolism
