# Supplementary material for: Edoxaban versus enoxaparin for the prevention of venous thromboembolism after total knee or hip arthroplasty: pooled analysis of coagulation biomarkers and primary efficacy and safety endpoints from two phase 3 trials
Source: Thromb J. 2016 Dec 1;14:48. doi: 10.1186/s12959-016-0121-1 (PMC5134224; doi:10.1186/s12959-016-0121-1)
Supplement: Additional file 1: Table S1. — Median and range of plasma concentrations of coagulation biomarkers at various time points after total knee or total hip arthroplasty. (DOCX 14 kb) [file 12959_2016_121_MOESM1_ESM.docx]

**Additional file 1: Table S1.** Median and range of plasma concentrations of coagulation biomarkers at various timepoints after total knee or total hip arthroplasty.

|  |  | **Preoperation** | | **Pretreatment** | | **Day 7^a^** | | **End of treatment**  **(day 11–14)^a^** | |
| --- | --- | --- | --- | --- | --- | --- | --- | --- | --- |
|  |  | **n** | **median**  **(min, max)** | **n** | **median**  **(min, max)** | **n** | **median**  **(min, max)** | **n** | **median**  **(min, max)** |
| D-dimer  (µg/mL) | Edoxaban | 535 | 0.460  (0.10, 6.78) | 535 | 5.49  (0.36, 155.3) | 532 | 3.99  (0.89, 14.09) | 528 | 4.86  (0.45, 16.35) |
|  | Enoxaparin | 527 | 0.48  (0.10, 8.36) | 527 | 5.83  (0.46, 142.9) | 480 | 5.08  (1.33, 24.35) | 472 | 5.58  (1.03, 22.86) |
| F_1+2_  (pmol/L) | Edoxaban | 535 | 243.0  (85, 2370) | 535 | 323.0  (86, 12800) | 532 | 327.0  (114, 2040) | 528 | 266.5  (93, 3040) |
|  | Enoxaparin | 527 | 242  (92, 2160) | 527 | 325.0  (96, 73300) | 480 | 422  (193, 1310) | 472 | 348.5  (117, 2130) |
| SFMC  (µg/mL) | Edoxaban | 535 | 3.00  (3.0, 250.0) | 535 | 13.20  (3.0, 250.0) | 532 | 3.30  (3.0, 102.0) | 528 | 4.20  (3.0, 157.0) |
|  | Enoxaparin | 527 | 3.00  (3.0, 94.6) | 527 | 12.90  (3.0, 250.0) | 480 | 4.10  (3.0, 163.0) | 472 | 5.00  (3.0, 129.0) |

^a^Predose

F_1+2_ = thrombin fragments 1+2; SD = standard deviation; SFMC = soluble fibrin monomer complex.
